# *PBRM1* deficiency oncogenic addiction is associated with activated AKT–mTOR signalling and aerobic glycolysis in clear cell renal cell carcinoma cells

**DOI:** 10.1111/jcmm.17418

**Published:** 2022-06-07

**Authors:** Yu Tang, Yan‐Hong Jin, Hu‐Li Li, Hui Xin, Jin‐Dong Chen, Xue‐Ying Li, You‐Fu Pan

**Affiliations:** ^1^ Department of Medical Genetics Zunyi Medical University Zunyi China; ^2^ Key Laboratory of Gene Detection and Treatment in Guizhou Province Zunyi China; ^3^ Department of Urology University of Rochester Medical Center Rochester New York USA; ^4^ Exploring Health LLC Guangzhou China

**Keywords:** aerobic glycolysis, AKT–mTOR signalling, ccRCC, HIF1α, *PBRM1(PB1)*

## Abstract

The *PBRM1 (PB1)* gene which encodes the specific subunit BAF180 of the PBAF SWI/SNF complex, is highly mutated (~ 40%) in clear cell renal cell carcinoma (ccRCC). However, its functions and impact on cell signalling are still not fully understood. Aerobic glycolysis, also known as the ‘Warburg Effect’, is a hallmark of cancer, whether *PB1* is involved in this metabolic shift in clear cell renal cell carcinoma remains unclear. Here, with established stable knockdown *PB1* cell lines, we performed functional assays to access the effects on 786‐O and SN12C cells. Based on the RNA‐seq data, we selected some genes encoding key glycolytic enzymes, including PFKP, ENO1, PKM and LDHA, and examined the expression levels. The AKT–mTOR signalling pathway activity and expression of HIF1α were also analysed. Our data demonstrate that PB1 deficiency promotes the proliferation, migration, Xenograft growth of 786‐O and SN12C cells. Notably, knockdown of PB1 activates AKT–mTOR signalling and increases the expression of key glycolytic enzymes at both mRNA and protein levels. Furthermore, we provide evidence that deficient *PB1* and hypoxic conditions exert a synergistic effect on HIF 1α expression and lactate production. Thus, our study provides novel insights into the roles of tumour suppressor *PB1* and suggests that the AKT–mTOR signalling pathway, as well as glycolysis, is a potential drug target for ccRCC patients with deficient *PB1*.

## 1 INTRODUCTION

Kidney cancer is a common tumour of the urinary system, with the rate of incidence accounting for 3%–5% of all tumours.^1^ In 2020, it was estimated to occur in 431,288 patients worldwide, resulting in 179,368 deaths.^2^ Kidney cancer has a variety of histopathological types and can be divided into at least10 types. Renal cell carcinoma (RCC) accounts for 90% of all kidney cancers, and clear cell renal cell carcinoma (ccRCC) is the most common histologic type of RCC.^3^

SWI/SNF (SWItch/sucrose non‐fermentable) is a subfamily of ATP‐dependent chromatin remodelling complexes, which is evolutionarily conserved from yeast to human and plays important roles in cell cycle, cell fate, cell death, metabolism, DNA repair, transcriptional regulation and tumorigenesis, among many others.^4^, ^5^, ^6^ In mammals, there are three distinct ATP‐dependent SWI /SNF complexes and each complex has an ATPase (BRM or BRG1) and 10–14 other common or specific subunits. The classic BAF (BRG1 or BRM‐associated factor) contains either ARID1A or ARID1B as a specific unit, while the PBAF (Polybromo‐associated BAF) contains PB1, ARID2, BRD7 and PHF10 as specific subunits, and the newly identified GBAF (GLTSCR1 or GLTSCR1L‐associated BAF) contains GLTSCR1 or GLTSCR1L and BRD9.^7^, ^8^ The genomic studies showed that genes encoding subunits of the SWI/SNF complexes are mutated in ~25% of human cancers,^9^ which implies the critical roles of SWI/SNF complexes in carcinogenesis and cancer development. *PBRM1 (PB1)* is the second most commonly mutated gene in ccRCC after *Vhl*, with a mutation rate that can be as high as 41%.^10^

Mouse models showed that the loss of *Vhl* was not sufficient to cause RCC tumour, while the double deletion of *Vhl* and *PB1* resulted in bilateral, multifocal, transplantable ccRCC tumours.^11^, ^12^ Moreover, the concurrent loss of *PB1* and *BAP1* drove ccRCC development from low grade to high grade.^13^, ^14^, ^15^ These data imply the critical tumour suppressor role of *PB1* or PBAF complex in carcinogenesis and development of ccRCC.

Metabolic reprogramming of tumour cells is a hallmark of cancer, and the metabolic shift to aerobic glycolysis (Warburg effect) is a feature for ccRCC.^16^, ^17^, ^18^ ccRCC is characterized by over‐deposition of glycogen and lipids in the cytoplasm,^19^ and thus, kidney cancer has long been considered as a metabolic disease.^20^, ^21^, ^22^ Furthermore, a correlation between an increase in metabolic activity and disease progression was observed in major renal cancer histological types.^18^, ^23^

There are lines of evidence showing that other mutated genes in ccRCC, such as *VHL*, *MET*, *FLCN*, *TSC1*, *TSC2*, *FH* and *SDH*, are involved in pathways that respond to metabolic stress or nutrient stimulation.^20^ Whether *PB1* plays a role in metabolic reprogramming such as aerobic glycolysis and how it functions remains unknown.^22^

Here, we carried out functional studies with stably knockdown cell lines of 786‐O and SN12C, in an attempt to understand the role of *PB1* in ccRCC cells and its molecular mechanisms in metabolic reprogramming. Our data provide new insights into the tumour‐suppressive role of *PB1* and also links with glycolysis, mTOR and HIF1α in ccRCC.

## 2 MATERIALS AND METHODS

### 2.1 Cell culture

Human kidney cancer cell lines 786‐O (Chinese Academy of Sciences Cell Bank) and SN12C (ATCC) were cultured on PRMI‐1640 medium (Hyclone) with 10% foetal bovine serum (FBS) (Biological Industries) and 1× penicillin–streptomycin (Solarbio), and the cells were cultured at 37°C in a humidified 5% CO_2_ incubator. Cobalt chloride (RHAWN) was used to induce chemical hypoxia.

### 2.2 Online databases

Gene Expression Profiling Interactive Analysis (GEPIA2) database and Lianchuan online platform were used for transcriptomic analysis. The server cBioPortal was used for co‐expression analysis between *PB1* and several well‐known genes encoding classic glycolytic enzymes. Kaplan–Meier Plotter was used to analyse the overall survival rate of ccRCC patients.

### 2.3 RNA extraction and quantitative real‐time PCR

Total RNA was extracted with Trizol (Solarbio), cDNA was synthesized by reverse transcription according to the manufacturer's instructions (Takara Holdings Inc.), quantitative real‐time PCR (qRT‐PCR) was performed by using SensiFast SYBR Kit (Bioline) on a CFX 96 instrument (Bio‐Rad) with the following parameter: 95°C for 5 min, 40 cycles of 95°C for 5 s and 58°C for 50 s. Finally, dissociation curves were run (65°C for 5 s and 95°C for 5 s) in order to identify specific products.

### 2.4 Western blot analysis

The cells were collected and lysed with RIPA buffer (Solarbio), in the presence of protease inhibitor (Roche #11836170001) and phenylmethylsulfonyl fluoride (PMSF) (Solarbio,#P0100). The protein concentration was quantified with the *BCA Protein Assay Kit* (Solarbio) following the manufacturer's instructions. After five volumes of proteins were mixed with one volume of 5× loading buffer and denatured at 100°C for 5 min, proteins were resolved on 10% SDS‐Page gels. PVDF membrane (Epizyme Biotech, China) was activated in 100% methanol and then blocked with 5% non‐fat milk in tris buffered saline with Tween 20 (TBST) for 2 h. The PVDF membrane was incubated with diluted primary antibody at 4°C overnight, washed with TBST three times and incubated with the diluted second antibody for 2 h at room temperature. The membranes were then washed with TBST, treated with ECL reagents and washed again with TBST three times. Protein bands were imaged by a Gel imaging system (Gel Doc XR, Bio‐Rad) and analysed with Image J software.

The following primary antibodies were used: BAF180/PB1 (Bethyl, #A301‐591A), Beta‐actin (HuaAn, #EM21002), PKM (Cell Signalling Technology, #D30G6), LDHA (HuaAn, #ET1608‐57), ENO1 (HuaAn, #ET1705‐56), mTOR (HuaAn, #ET1608‐5), p‐mTOR (Cell Signalling Technology, #D9C2), AKT (HuaAn, #ET1609‐47), p‐AKT (HuaAn, #ET1607‐73) and HIF1α (Abcam, #ab2185). The horseradish peroxidase‐conjugated secondary antibodies, Goat anti‐Rabbit or Goat anti‐Mouse (Proteintech, #SA00001‐1/2) were also used in this study. The experiment was performed in duplicate or triplicate, and the representative blots are shown.

### 2.5 siRNAs transfection

Small interfering RNAs (siControl, siPB1‐1, and siPB1‐2) were designed and synthesized by Genepharma (Shanghai). Cells were digested with trypsin–EDTA and 6 × 10^5^ cells were seeded in wells of a 6‐well plate. The following day cells were transfected using the RNA‐Mate (Genepharma) following the manufacturer's instructions. The cells were harvested 72 h later and used for Western blot analysis or RNA extraction.

### 2.6 Short hairpin RNA (shRNA) lentivirus transduction

ShRNA lentiviruses (shNC, shPB1‐1 and shPB1‐2) were designed and packaged by Shanghai Genepharma. Lentivirus transduction was performed according to the manufacturer's instructions. In brief, the cells were digested and 1 × 105 cells were seeded in each well of the 6‐well plate at 37°C and 5% CO_2_ overnight. The following day, shRNA lentiviruses were used to transduce the cells (MOI = 10), in the presence of 5 μg/ml of polybrene (Sigma, #TR‐1003) in the medium. Stable clones were selected by adding puromycin dihydrochloride (Solarbio) at 3 μg/ml for 786‐O and 2 μg/ml for SN12C, respectively. qRT‐PCR and Western blot were used to examine the knockdown efficiency.

### 2.7 CCK‐8 cell proliferation experiment

After the cells were digested with 0.05% trypsin–EDTA, they were suspended in 1640 medium supplemented with 10% FBS and 1× penicillin/streptomycin solution. 2 × 10^3^ cells per well for 786‐O and 3 × 10^3^ cells per well for SN12C were reseeded in 96‐well plates (BKMAN), respectively. The cells were kept at 37°C in a humidified 5% CO_2_ incubator. At indicated time points (Days 0, 1, 2, 3 and 4), 10 μl of CCK8 reagent (Beyotime) was added to each well of a 96‐well plate, samples were incubated at 37°C for 2 h followed by measuring the absorbance at 450 nm using a multifunctional microplate reader (SpectraMax M2e, Molecular Devices).

### 2.8 Cell colony formation assay

Cell colony formation assay was performed basically as Franken et al described.^24^ In brief, after the cells were digested and resuspended as mentioned above, 2 × 10^3^ cells per well for 786‐O and 4 × 10^3^ cells per well for SN12C were reseeded in 6‐well plates. The cells were grown for 12 days, and the medium was changed every 3 days. Then, the medium was removed, the clones were washed with phosphate‐buffered saline (PBS) and fixed with 4% paraformaldehyde for 20 minutes. After the clones were stained with 1% crystal violet for 40 min and washed with PBS, the images were taken for statistical analysis.

### 2.9 Wound healing assay

The cells were cultured in a 6‐well plate and the wound healing assay was carried out when the cell density reached 90%. The cells were scratched evenly with a 200 μl pipette tip to simulate a wound. The floating cells were washed off and 1640 medium supplemented with 2% FBS was added to each well. The images were acquired using an inverted Leica DMIRB microscope at 0, 24 and 48 h time points.

### 2.10 Transwell migration experiment

The transwell migration experiment was carried out in 24‐well transwell chambers (Corning). After the cells were digested with 0.05% trypsin–EDTA, the cells were suspended in serum‐free RPMI 1640 medium. 200 μl of RPMI (2.0 × 10^4^ cells for 786‐O and 4.0 × 10^4^ cells for SN12C) was added in the upper chamber, while 800 μl of RPMI containing 20% FBS was added to the lower chamber.

The upper chamber was removed after culturing the cells for 21 h. Excess cells in the upper chamber were gently wiped away with a cotton swab and the chamber was washed twice with PBS, then, the cells were fixed with 4% paraformaldehyde (Biosharp) for 20 min. After the cells were stained with 1% crystal violet solution for 1 h at room temperature and gently rinsed with distilled water, five fields were randomly selected and counted under an inverted microscope. The experiment was performed in triplicate.

### 2.11 Subcutaneous tumour formation in nude mice

The tumour formation assay was conducted in compliance with animal protocols approved by the Laboratory Animal Ethics Committee at Zunyi Medical University. In total, 15 male BALB/c‐Nu mice (~6 weeks old) were purchased from the Animal Center of Zunyi Medical University. Each mouse was subcutaneously inoculated with 1 × 10^7^ cells (either shControl or shPB1‐1 or shPB1‐2) suspended in 100 μl PBS using a 1 ml syringe. The body weight and tumour size were monitored every 5 days. Mice were sacrificed after 4 weeks of inoculation. The tumours were weighed and analysed.

### 2.12 Measurement of lactate concentration

The concentration of lactic acid was measured using a LA assay kit (Solarbio, #BC2230) according to the manufacturers' instructions. In brief, after the cells were scraped off and resuspended, 1 × 10^6^ cells were homogenized at 4°C with an ultrasonic sonicator (CIENTZ). Following centrifugation at 10,000 *g* at 4°C for 10 min, the individual supernatant and reaction reagent was mixed and kept at 37°C for 15 min before transferring to a 96‐well plate. Finally, the absorbance at 450 nm was measured using a multifunctional microplate reader (SpectraMax M2e, Molecular Devices).

## 3 RESULTS

### 3.1 Knockdown of *PB1* promotes the migration, invasion and xenograft growth of ccRCC cells

**FIGURE 1:**
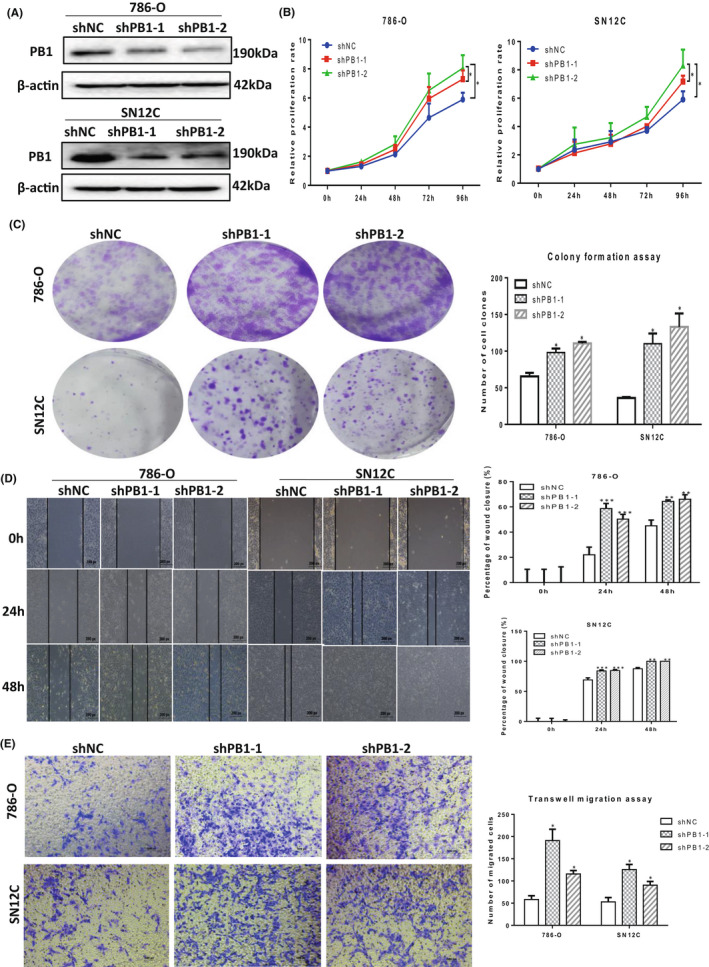
Knockdown of *PB1* enhances the proliferation, migration and invasion capabilities of 786‐O and SN12C cells. (A)Western blot results showing remarkable reduction of *PB1* expression. (B) CCK8 assay showing knockdown of *PB1* significantly promoted the proliferation rate of ccRCC cells. (C) The colony formation assay showing the colony formation capability for 786‐O and SN12C cells. (D) The wound‐healing assay showing that the migration capability for 786‐O and SN12C cells. (E) The transwell assay showing the migration capability for 786‐O and SN12C cells. The representative images (left)) and the quantitative results of independent duplicate (right) were shown in C, D and E. (*p < 0.05; **, *p* < 0.01; **, *p* < 0.001)

ShRNA lentiviruses (shControl, shPB1‐1 and shPB1‐2) were used to infect 786‐O and SN12C cell lines. The knockdown efficiency was examined by Western blotting which showed a remarkable drop at the protein level for both shRNAs (shPB1‐1 & shPB1‐2) (Figure 1A). Then, the two ccRCC cell lines were used for further studies.

We performed the CCK8 assay to examine the cells' proliferation capability upon stably knockdown of PB1. There was no significant difference between shControl group and shPB1 group until Day 4 for both cell lines (Figure 1B).

The colony formation assay was performed to evaluate the abilities of 786‐O and SN12C cells to survive and reproduce to form colonies upon knockdown of *PB1*. There were 65.5 ± 4.9, 98.0 ± 5.7 and 110.5 ± 2.1 colonies for the 786‐O shControl group, shPB1‐1 and shPB1‐2 group, respectively. Similarly, there were 36 ± 1.4, 110 ± 14.1 and 130 ± 18.4 colonies for the SN12C shControl group, shPB1‐1 and shPB1‐2 group, respectively (Figure 1C). The wound‐healing assay showed that the migration capability for 786‐O and SN12C cell lines was enhanced significantly upon the knockdown of *PB1* (Figure 1D). Similar results were observed when the transwell assay was performed for both cell lines (Figure 1E).

**FIGURE 2:**
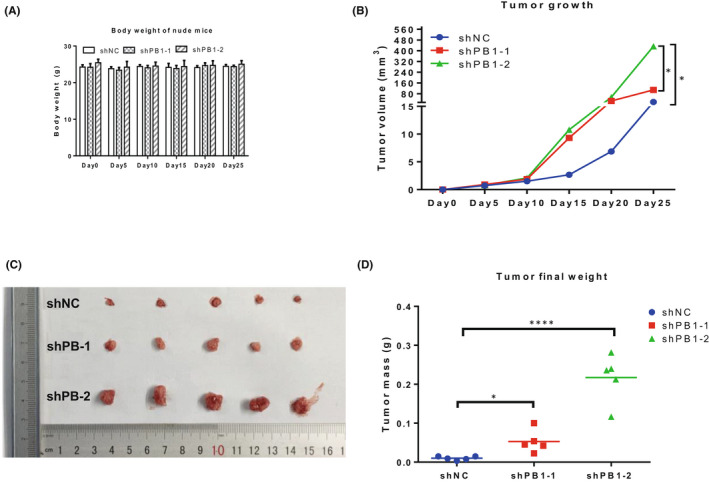
Knockdown of *PB1* promotes the xenograft growth. (A)The mice were monitored every 5 days for 4 weeks and the body weights were measured. (B) Tumour volume in nude mice. (C) The xenografts were taken out after growing in nude mice for 25 days. (D) The final tumour weight was measured. (*p < 0.05; **, *p* < 0.01; **p < 0.001)

Furthermore, our subcutaneous tumour formation assay showed that knockdown of PB1 in 786‐O cells promotes the xenograft growth significantly compared with the control group (Figure 2A–D).

### 3.2 The deficient *PB1* activates AKT/mTOR signalling pathway

**FIGURE 3:**
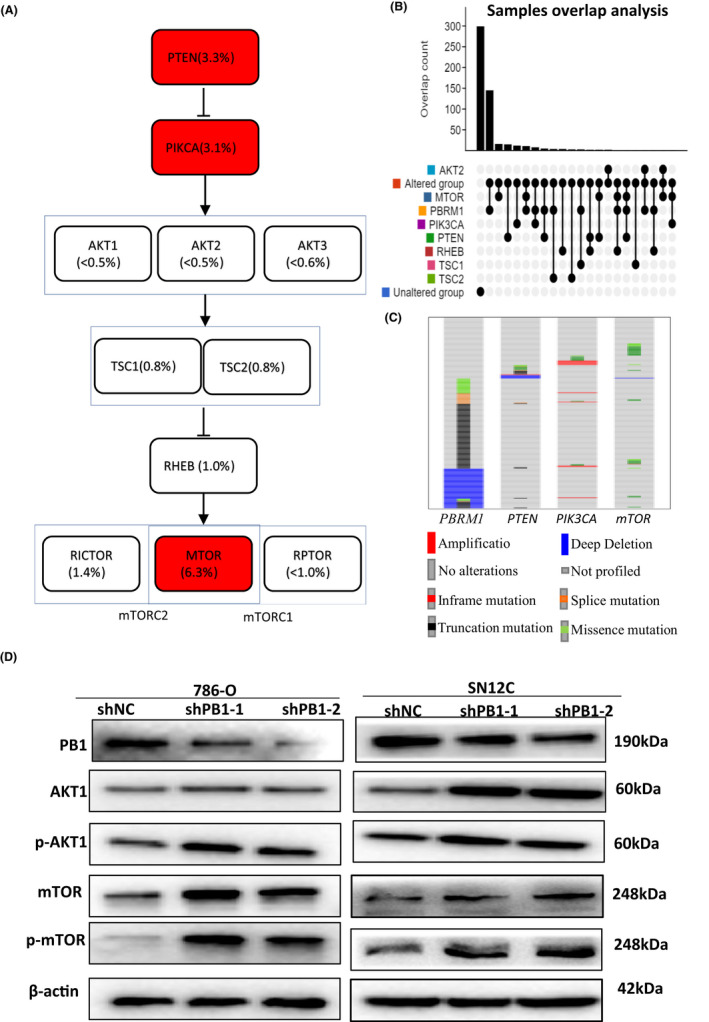
Deficient *PB1* activates AKT–mTOR pathway in 786‐O and SN12C cells. (A) Analysis of TCGA data (Firehose Legacy) showing the key AKT–mTOR signalling players including PTEN, PIK3CA, AKT2, TSC1, TSC2, RHEB, MTOR are mutated; red box indicates the mutation rate of the gene is greater than 3%. (B) Overlap analysis among PTEN,PIK3CA,AKT2,TSC1,TSC2,RHEB,MTOR mutations with 528 RCC samples. (C) Screen shot showing comparisons among PBRM1, PTEN, PIK3CA and mTOR. Each horizontal bar represents a sample. Colour bars represent samples with genetic alterations and grey bars are samples not altered genetically. (D)Western blot analyses of AKT1, mTOR, p‐AKT1 and p‐mTOR in 786‐O and SN12C cells

The AKT/ mTOR pathway is one of the most activated signalling pathways in ccRCC.^22^, ^25^ Our analysis showed that the key AKT–mTOR signalling players including *PTEN*, *PIK3CA*, *AKT2*, *TSC1*, *TSC2*, *RHEB and MTOR* were mutated(Figue 3A). However, only a small percentage of *MTOR* mutations (7.97%) co‐occurs in samples with *PB1* mutations. This is also true for other genes of the AKT–mTOR signalling pathway (Figure 3B,C). Since these mutations seem to be mutually exclusive, we speculated that deficient *PB1* may activate the AKT–mTOR signalling pathway. To test this hypothesis, we examined the protein level of Akt, p‐AKT, mTOR and p‐mTOR. The Western blot results demonstrated that deficient *PB1* did increase the levels of Akt and mTOR in both 786‐O and SN12C cells, as well as their phosphorylated forms (Figure 3C).

### 3.3 Deficient *PB1* increase the protein levels of glycolytic enzymes

**FIGURE 4:**
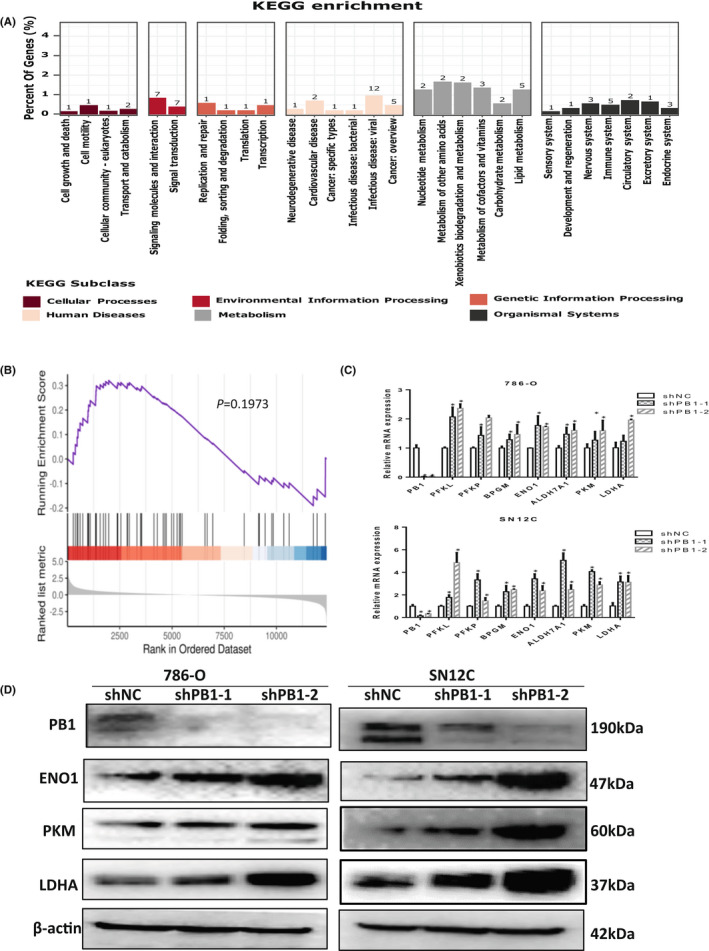
Knockdown of *PB1* increases the expression levels of key glycolytic enzymes. (A) RNAseq data showing enriched KEGG terms in 786‐O cells. (B) GSE analysis showing that the glycolysis pathway was slightly enriched (*p* = 0.1973). (C) QRT–PCR analysis from two independent duplicates showing the mRNA levels of the selected gene. (D) Representative results of a Western blot showing the protein levels of key glycolytic enzymes. (*p < 0.05)

Previously, we generated RNA‐seq data in 786‐O cells with siRNA targeting *PB1*, in order to understand what processes or pathways were regulated by *PB1* at the transcriptional level. Our data indicate a number of KEGG terms were enriched, including cell growth and death, cell motility, replication and cell cycle control (Figure 4A). Interestingly, we noticed that these dysregulated genes were also involved in metabolism such as carbohydrate metabolism and lipid metabolism. GSE analysis showed that glycolysis was slightly enriched though not significantly (*p* = 0.1973) (Figure 4B). To further clarify this, we performed qRT‐PCR to examine the expression of selected genes that encode some well‐known glycolytic enzymes, such as phosphofructokinase (PFKP, PFKL), glycoenolase (ENO1), pyruvate kinase (PKM), lactate dehydrogenase (LDHA) and aldehyde dehydrogenase (ALDH7A1). These genes were all upregulated at the mRNA level upon knockdown of *PB1*, which was consistent with RNAseq data (Figure 4C). The Western blot results further confirmed that the protein level of PFKP, ENO1 PKM and LDHA all increased in both cell lines upon knockdown of *PB1* (Figure 4D).

**FIGURE 5:**
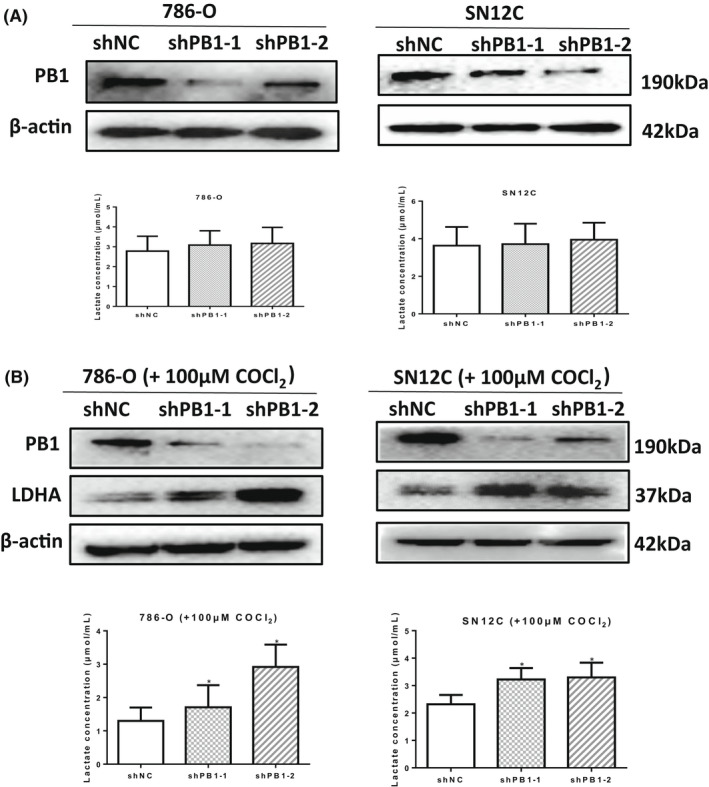
Depletion of *PB1* promotes lactate production under hypoxic conditions. (A) Western blot analysis showing the protein levels of PB1 (top) and measurement of the lactate concentration, under normoxic conditions. (B) Western blot analysis showing the protein levels of LDHA (top) and measurement of the lactate concentration, under hypoxic conditions induced by CoCl2 (100 μM). Experiments were performed in independent duplicates. (*p < 0.05)

To further confirm that the glycolysis metabolic pathway is activated by deficient *PB1*, we next measured the concentration of lactate acid, the final product of glycolysis. To our surprise, there was no significant difference between the shControl group and the shPB1 group (Figure 5A).

We speculated that the remarkable activation of glycolysis may be a synergistic effect of deficient *PB1* combined with hypoxic conditions. Then, we treated the cells with CoCl_2_ at a final concentration of 100 μM. Indeed, under hypoxic conditions induced by CoCl_2_, the protein level of LDHA and the concentration of lactate in SN12C cells were both dramatically increased in shPB1 groups compared with shControl group (Figure 5B).

### 3.4 Deficient *PB1* activates the glycolysis signalling independent of **HIF1α**

**FIGURE 6:**
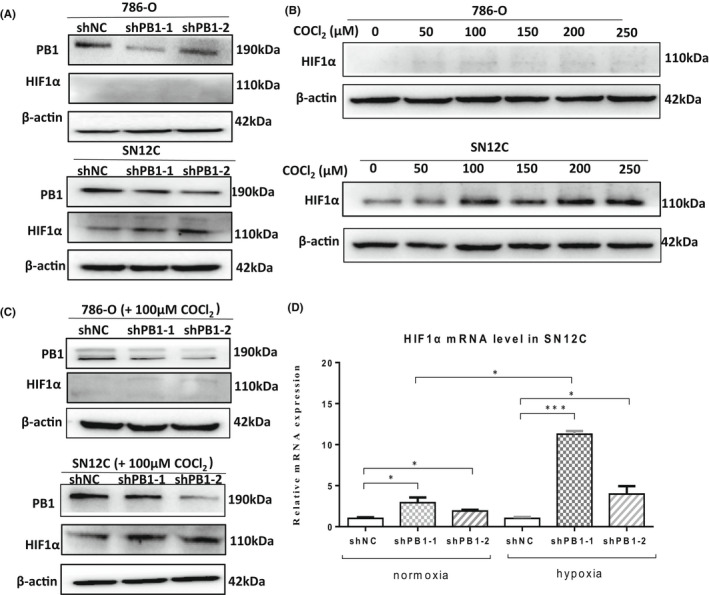
Depletion of *PB1* further increases the protein level of HIF1α under hypoxic conditions in SN12C cells. (A) Western blot analysis showing the protein levels of HIF1α under normal conditions in 786‐O and SN12C cells. (B) Western blot analysis showing HIF1α levels when cells were treated with different concentrations of CoCl_2_. (C) Western blot analysis showing the protein levels of HIF1α under hypoxic conditions (100 μM COCl_2_). (D) QRT–PCR analysis showing the mRNA levels under normoxic and hypoxic conditions. (*p < 0.05, ***p < 0.001)

Hypoxia‐inducible factor HIF1α is a key player in ccRCC which can be accumulated due to *Vhl* loss. To investigate if deficient *PB1* also increases the expression of HIF1α, we examined the protein level of HIF1α and found that there was no band detected in 786‐O cells, which is consistent to report that HIF1α is lost in 786‐O cells.^26^ However, the HIF1α protein level did increase in SN12C cells under normal conditions (Figure 6A). This suggests that the activation of glycolysis signalling in 786‐O cells is independent of HIF1α, and this may also be true for SN12C cells.

To further investigate if deficient *PB1* combined with hypoxic conditions exert synergistic effect on HIF1α expression, we examined the HIF1α level under hypoxic conditions induced by CoCl_2_. We observed that HIF1α could be induced by CoCl_2_ at different concentrations (ranging from 50‐250 μM) in SN12C cells (but not in 786‐O cells) (Figure 6B). Moreover, the synergistic effect on the HIF1α protein level increase was clearly observed in SN12C cells under hypoxic conditions (Figure 6C) and at mRNA level (Figure 6D).

### 3.5 *PB1* level is negatively correlated with the expression of key glycolytic enzymes in clinic samples

**FIGURE 7:**
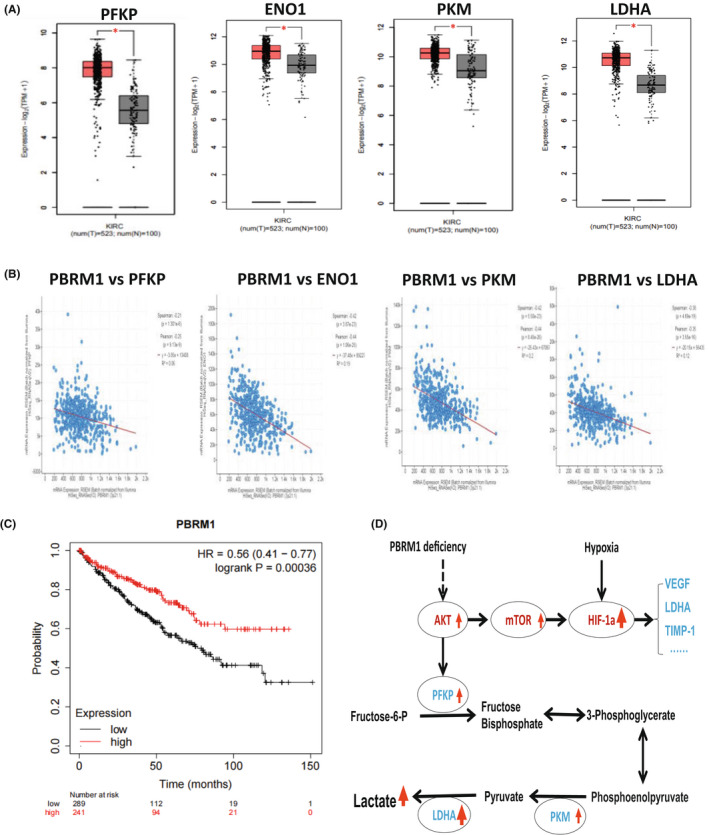
*PB1* is negatively correlated with the mRNA levels of key glycolytic enzymes in clinic ccRCC samples. (A) TCGA data analysis showing the expression levels of *PFKP*, *ENO1*, *PKM*, *LDHA* in ccRCC tumours and the matched adjacent normal controls. (B) Co‐expression analysis of selected genes and *PB1*. (C) Overall survival analysis of ccRCC patients with high‐ or low‐level PB1. (D) The suggested mechanism shows that the AKT–mTOR and glycolysis signalling pathways could be activated by deficient *PB1*, HIF1α level could also be increased by deficient *PB1*, implying deficient *PB1* together with hypoxic conditions exerting a synergetic effect on HIF1α expression. (*p < 0.05)

Next, we examined the expression of key enzymes catalysing glycolysis such as PFKP, ENO1, PKM and LDHA in ccRCC tumours and the matched controls. The analysis showed that these genes were all highly expressed in tumour samples compared with adjacent normal controls (Figure 7A). We further analysed the relationship of *PB1* expression with these enzyme genes. As expected, low‐level *PB1* was significantly correlated with high‐level *PFKP*, *ENO1*, *PKM and LDHA* (Figure 7B). The overall survival analysis demonstrates that deficient or low‐level *PB1* was significantly correlated with worse outcomes, while high‐level *PB1* could prolong the patient's life expectancy (Figure 7C).

Based on these data, we proposed that given *HIF1α* expression is induced under hypoxic conditions, deficient *PB1* not only activates AKT–mTOR and the glycolysis signalling pathways but also exerts a synergetic effect on HI1a expression, which collectively promote ccRCC tumour growth (Figure 7D).

## 4 DISCUSSION

In order to understand the functional role of *PB1* and the mechanisms, we firstly performed the CCK8 assay, colony formation assay and then, migration assay using the stable cell lines with reduced *PB1* expression. These functional assays demonstrate that *PB1* does function as a tumour suppressor and promotes the proliferation and migration of ccRCC cells. Moreover, the nude mice assay showed that deficient *PB1* promotes xenograft tumour growth in vivo.

Our immunofluorescence assay clearly showed that PB1 is localized in the nuclei, which is consistent to its role functioning in chromatin remodelling process and gene transcriptional control (Figure S1). To investigate the dysregulated pathways upon knockdown of *PB1*, we firstly focused on the AKT–mTOR signalling pathway, because AKT–mTOR is one of the most disturbed signalling pathways and a large number of ccRCC samples harbouring *PB1* mutations do not have mutations of other key genes of AKT–mTOR signalling pathway such as *PTEN,PIK3CA,AKT2,TSC1,TSC2,RHEB* and *MTOR*. We examined the expression of AKT and mTOR, as well as phosphorylated p‐AKT and p‐mTOR. The data clearly showed that deficient *PB1* did activate the AKT‐ mTOR signalling pathway. This finding uncovers a new alternative way to activate mTOR signalling by deficient *PB1* other than mutations that occurred in *mTOR* and related genes in ccRCC cells. Considering the high mutation rate of *PB1*, this also implies that the AKT‐mTOR pathway may play a more critical role than we should have expected in ccRCC development.

Aerobic glycolysis, also known as the ‘Warburg Effect’, is a hallmark of cancer where the cancer cells rely on glycolysis for growth even in the presence of oxygen. It is unclear whether PB1 plays a role in this metabolic shift. Then, we speculated that the glycolysis pathway may be activated by deficient *PB1*. Our RNAseq data showed some genes encoding glycolytic enzymes were upregulated; however, the glycolysis pathway was only slightly enriched in GSE analysis (*p* = 0.1973). We suspected that this might be due to limitations of in vivo normoxic conditions.

We examined the expression levels of enzymes playing critical roles in aerobic glycolysis and the activity of lactate dehydrogenase. We did observe an increase of key glycolytic enzymes (such as PFKP, ENO1 PKM and LDHA) under normoxic conditions. This increase was also observed in ccRCC clinic samples compared with the normal controls. However, we did not see significant difference on the lactate production between the two groups.

In order to understand the role of *PB1* in metabolic shift and if there is synergistic effect under hypoxic conditions, we induced hypoxia in vitro via treating the cells with CoCl_2._ Indeed, we observed an increase in the production of lactate in the *PB1* deficient group under hypoxic conditions. Furthermore, this enhanced glycolysis was accompanied by elevated HIF1α level, which suggests there is a synergistic effect possibly exerted by deficient *PB1* and further upregulation of HIF1α under hypoxic conditions.

Both *Vhl* and *PB1* are tumour suppressor genes highly mutated in ccRCC. Based on mouse model studies, loss of *Vhl* alone cannot cause kidney tumour, and only when both genes are inactivated the tumour may form.^11^, ^12^ It is well known that the HIF1α level can be elevated by loss of *Vhl*, our finding that HIF1α level can also be elevated by deficient *PB1* provides a new explanation for why angiogenesis is featured in ccRCC samples with wild‐type *Vhl*. It is also consistent to the observation that expression of full‐length HIF1α and PB1protein seems to be mutually exclusive in most ccRCC cell lines and in some primary tumours.^26^

## 5 CONCLUSION

Taken together, our study demonstrates that *PB1* does function as a tumour suppressor, and *PB1* deficiency oncogenic addiction is associated with the activated AKT–mTOR and glycolysis signalling pathways. The HIF1α expression level can also be elevated by deficient *PB1*. Our data suggest that future translational studies should pay more attention to the AKT–mTOR, glycolysis and angiogenesis signalling pathways in *PB1* deficient ccRCC patients, as well as other tumour types with *PB1* deficiency.

## AUTHOR CONTRIBUTIONS

**Yu Tang:** Formal analysis (equal); investigation (equal); writing – original draft (equal). **Yan‐Hong Jin:** Formal analysis (equal); investigation (equal); validation (equal). **Hu‐Li Li:** Validation (equal). **Hui Xin:** Validation (equal). **Jin‐Dong Chen:** Resources (equal); writing – review and editing (supporting). **Xue‐Ying Li:** Project administration (supporting); resources (equal). **You‐Fu Pan:** Conceptualization (lead); formal analysis (lead); funding acquisition (lead); investigation (lead); methodology (lead); project administration (lead); supervision (lead); writing – original draft (lead); writing – review and editing (lead).

## CONFLICT OF INTEREST

The authors declare no conflict of interest.

## DATA ANALYSIS

We used Photoshop 6.0 to crop the images and used Graphpad Prism 6 and SPSS V 16.0 to plot the data. For statistical analysis, the results were all expressed as $\bar{x}$ ± SD. The two‐tailed Student's *t‐*test was used to examine the significance level.

## Supporting information


Figure S1

## Data Availability

The expression profiles were deposited to NCBI under BioProject accession No.PRJNA771094.
