# Supplementary figures and images for: PBRM1 deficiency oncogenic addiction is associated with activated AKT–mTOR signalling and aerobic glycolysis in clear cell renal cell carcinoma cells
Source: J Cell Mol Med. 2022 Jun 7;26(14):3837–49. doi: 10.1111/jcmm.17418 (PMC9279584; doi:10.1111/jcmm.17418)

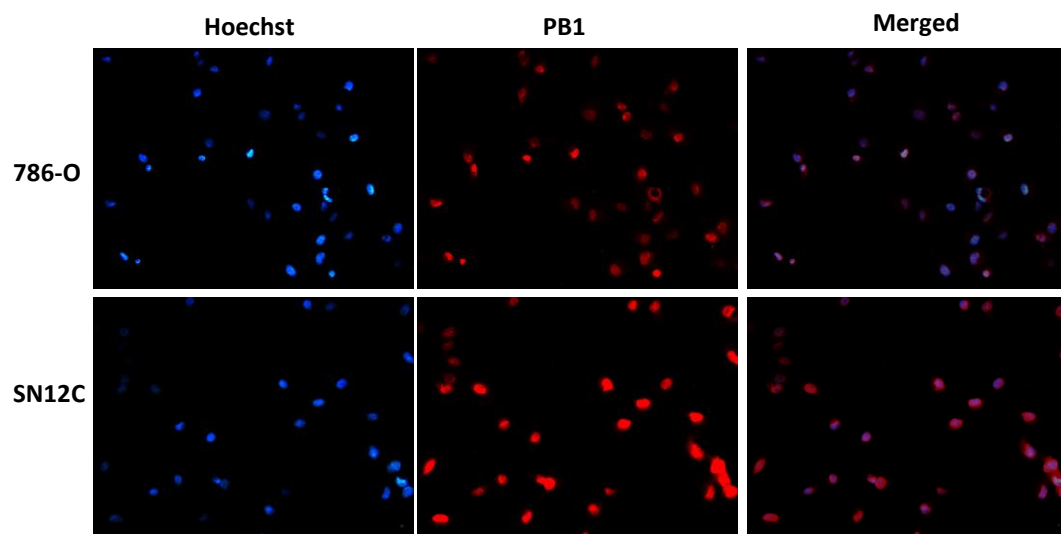

Supplement: Supplementary file 1 — Figure S1 [file JCMM-26-3837-s001.pdf]
